# Quantitative Estimation of the Equivalent Radiation Dose Escalation using Radiofrequency Hyperthermia in Mouse Xenograft Models of Human Lung Cancer

**DOI:** 10.1038/s41598-019-40595-6

**Published:** 2019-03-08

**Authors:** Bibin Prasad, Subin Kim, Woong Cho, Jung Kyung Kim, Young A. Kim, Suzy Kim, Hong Gyun Wu

**Affiliations:** 1 Department of Mechanical Engineering, Graduate School, Kookmin University, Seoul, 02707 Republic of Korea; 2 Department of Radiation Oncology, SMG-Seoul National University Boramae Medical Center, Seoul, 07061 Republic of Korea; 3 School of Mechanical Engineering and Department of Integrative Biomedical Science and Engineering, Graduate School, Kookmin University, Seoul, 02707 Republic of Korea; 4 Department of Pathology, SMG-Seoul National University Boramae Medical Center, Seoul, 07061 Republic of Korea; 5 Department of Radiation Oncology and Cancer Research Institute, Seoul National University College of Medicine, Seoul, 03080 Republic of Korea; 6 Present Address: Department of Radiation Oncology, SMG-Seoul National University Boramae Medical Center, Seoul, 07061 Republic of Korea

## Abstract

Hyperthermia is a potent radiosensitizer, and its effect varies according to the different types of cancer cells. In the present study, the radiosensitizing effect of hyperthermia on lung cancer cell lines A549 and NCI-H1299 was determined based on the equivalent radiation dose escalation. *In vitro* cell experiments were conducted using lung cancer cell lines A549 and NCI-H1299 to determine thermal radiosensitivity. *In vivo* experiments were conducted using mouse heterotopic xenograft models to determine the treatment response and increase in the temperature of tumors using a 13.56 MHz radiofrequency (RF) hyperthermia device. Using the *α* and *β* values of the linear–quadratic equations of cell survival curves, numerical simulations were performed to calculate the equivalent radiation dose escalations. The dielectric properties of tumors were measured, and their effect on the calculated equivalent radiation dose was analyzed. Hyperthermia increased the equivalent radiation dose of lung cancer xenografts and a higher escalation was found in NCI-H1299 cells compared with that observed in A549 cells. An underestimation of the calculated equivalent radiation dose was observed when the dielectric property of the tumor was varied. This study may contribute to the effective planning of thermoradiotherapy in clinics.

## Introduction

Mild hyperthermia ranging from 41 °C to 45 °C, combined with radiation therapy (RT), has shown excellent clinical results^1,2^. Hyperthermia is normally used once or twice per week during RT. The combined effects of radiation and hyperthermia may be quantified in terms of equivalent dose, where the radiation dose distribution with hyperthermia is converted to an equivalent radiation dose^3–5^. Biological modeling is often applied to calculate the equivalent radiation dose distributions. The linear–quadratic (LQ) model, defining the number of lethal lesions as the sum of the lethal lesions produced from a single radiation track and those produced from two radiation tracks, is widely used for this purpose^6–8^.

Radiosensitization caused by hyperthermia may be modeled as a temperature-dependent variation of the radiosensitivity parameters *α* and *β* in the LQ model and the equivalent radiation dose model combining the effects of hyperthermia. *α* and *β* vary according to the different types of cancer cells and radiation^9–11^. Thermoradiotherapy planning using the LQ model incorporates the DNA repair inhibition mechanism because it is the dominant form of radiosensitization using hyperthermia^5,12^. Other mechanisms, such as direct cell killing and reoxygenation, should also be considered in more advanced and sophisticated thermoradiotherapy planning to achieve improved treatment outcomes^12^.

Linear and exponential mathematical models determining the thermal sensitivity of cancer cell lines have been reported previously^3^, and an escalation of the equivalent radiation dose was determined for patients with cervical and prostate cancer receiving simultaneous therapy with radiation and hyperthermia^3,5^. Equivalent radiation dose escalations of >10 and 7–11 Gy were reported previously in prostate^3^ and cervical cancer^5^. These studies showed a useful method to compare the effectiveness of hyperthermia in RT and design/guide the dose escalation using hyperthermia in clinical studies.

**Figure 1:**
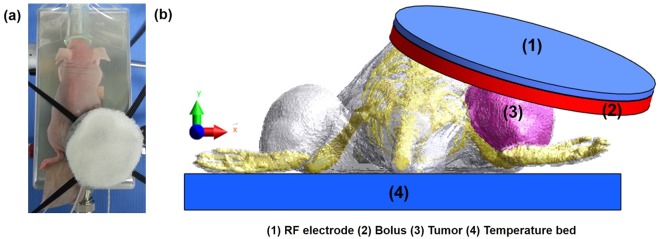
Mouse xenograft model used in this study: (**a**) Experimental mouse model for the measurement of temperature; (**b**) Computational mouse used for numerical simulation.

The objective of the present study was to determine the equivalent radiation dose escalation in lung cancer cell lines A549 and NCI-H1299. *In vitro* experiments were performed to determine the thermal radiosensitivity parameters and propose a modified linear model of temperature dependency of *α* in the treatment of lung cancer*. In vivo* experiments and numerical simulations were performed using a mouse xenograft model (Fig. 1(a,b)) to evaluate the effect of varying dielectric properties of tumors and confirm the equivalent radiation dose escalation.

## Results

**Figure 2:**
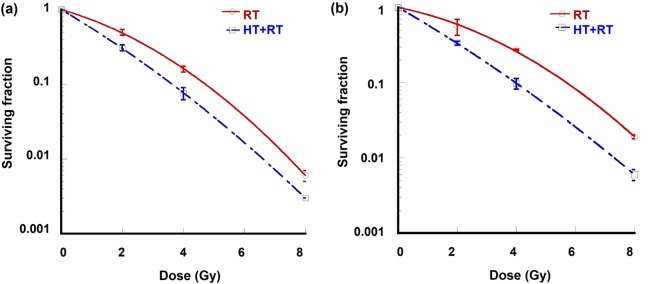
(**a**,**b**) Clonogenic cell survival curves of (**a**) A549 and (**b**) NCI-H1299 cells. Surviving fraction of cells treated with hyperthermia (42 °C for 30 min) prior to radiation were compared with those of radiation alone. *Points*: mean for three independent experiments, *bars*: standard errors.

Figure 2(a,b) Depicts the logarithmic fractional cell survival of A549 and NCI-H1299 cells treated with RT alone and combined with hyperthermia therapy (HT). The addition of HT to RT increased the fraction of cell death and enhanced the radiosensitivity of NCI-H1299 cells and A549 cells. The addition of HT treatment at 42 °C to RT (2–8 Gy) enhanced the  linear parameter (*α*) to 0.53 from 0.23 Gy^−1^ for A549 and to 0.51 from 0.24 Gy^−1^ for NCI-H1299, respectively, which elucidates the radiosensitizing effect of HT on lung cancer cell lines. The average values of *α* and *β* of the cells treated with RT alone or with combined HT and RT are detailed in Table 1.

**Table 1:** Effect of hyperthermia on the average values of *α* and *β*, with radiation therapy alone and combined with hyperthermia therapy dose.

Lung cancer cell line	Treatment	*α* (Gy^−1^)	*β* (Gy^−2^)
A549	RT	0.2398	0.0546
	30 min 42 °C + RT	0.5318	0.0283
NCI-H1299	RT	0.2415	0.0229
	30 min 42 °C + RT	0.5141	0.0163

**Figure 3:**
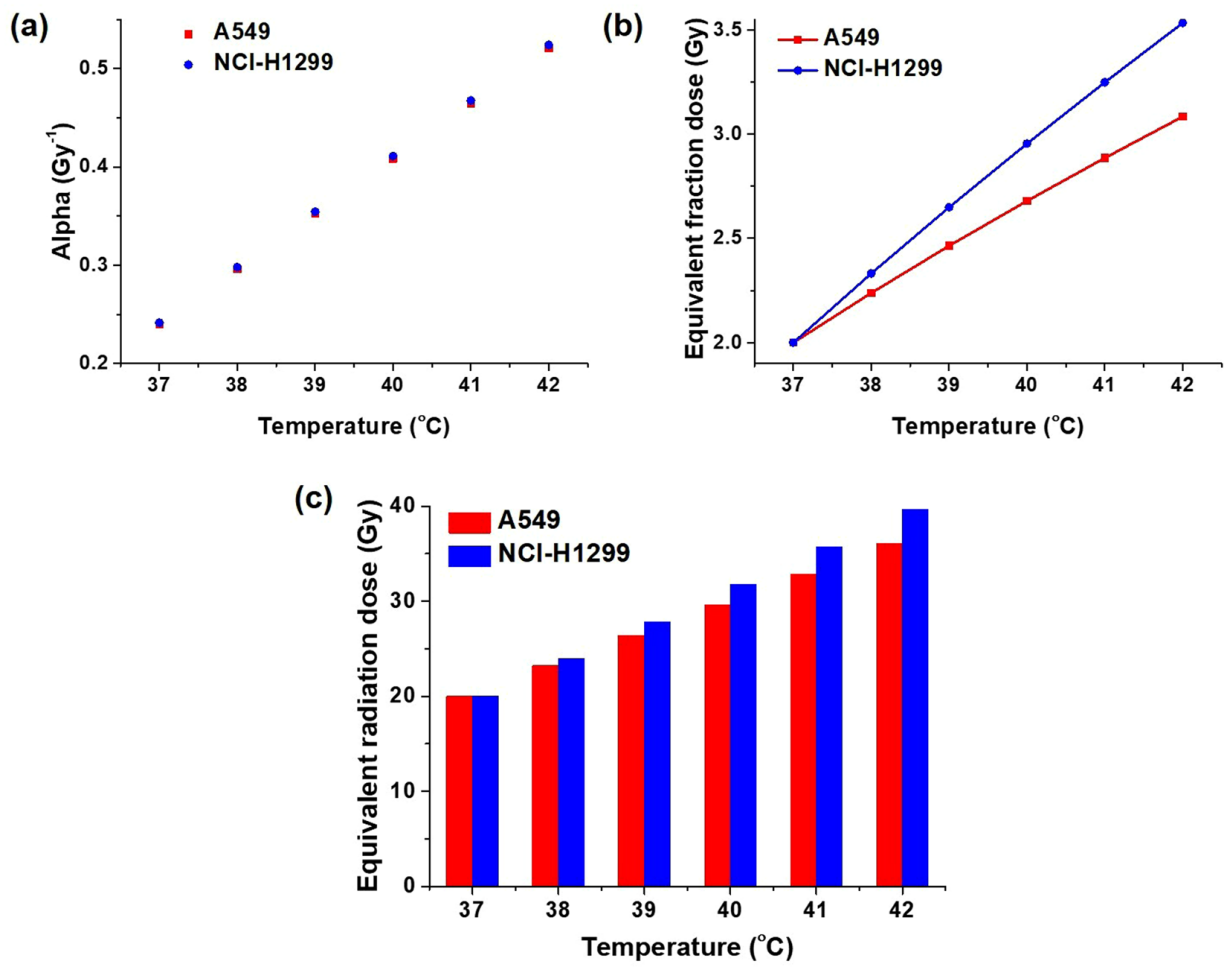
Analysis of equivalent radiation dose: (**a**) Temperature dependency of the radiosensitivity parameter *α* of lung cancer cell lines A549 and NCI-H1299; (**b**) Equivalent fraction dose escalation for A549 cells and NCI-H1299 cells with respect to temperature; (**c**) Comparison of the equivalent radiation dose escalation for A549 cells and NCI-H1299 cells with respect to temperature.

The temperature dependency of *α* in the lung cancer cell lines A549 and NCI-H1299 is illustrated in Fig. 3(a) based on the linear model calculation (equation 6) and input conditions in Table 2. It was observed that, in response to an increase in temperature from 37 °C to 42 °C, *α* tended to increase linearly from 0.2 to 0.5 Gy^−1^ for both A549 cells and NCI-H1299 cells. The increased *α* enhanced the radiation dose fraction from 2 to 3.08 Gy for A549 cells and 3.53 Gy for NCI-H1299 cells, as shown in Fig. 3(b). If a mouse xenograft was treated with 10 fractions of radiation (2 Gy/fraction), the equivalent radiation dose would have been 36.07 Gy for A549 cells and 39.66 Gy for NCI-H1299 cells at 42 °C, as shown in Fig. 3(c). We found that hyperthermia increased the effect of the radiation dose of 16.07 Gy for A549 cells and 19.66 Gy for NCI-H1299 cells. The enhancement of the radiation dose effect by HT was greater in NCI-H1299 cells compared with that observed in A549 cells. The temperature-dependent comparison of the equivalent radiation dose escalation is shown in Fig. 3(c).

**Table 2:** Input conditions for the calculation of the equivalent radiation dose.

Parameters	Input conditions
*D*	20 Gy
*d*	2 Gy
*α*_37_ (A549)	0.2398 Gy^−1^
*β*_37_ (A549)	0.0546 Gy^−2^
*α*_37_ (NCI-H1299)	0.2415 Gy^−1^
*β*_37_ (NCI-H1299)	0.0229 Gy^−2^
*G*	0.1

**Figure 4:**
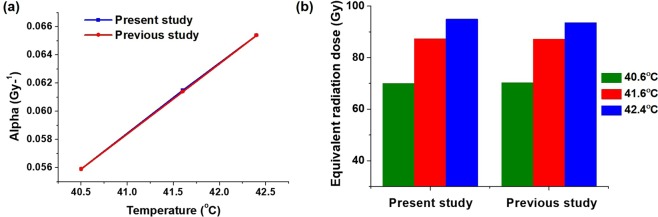
Numerical validation for the temperature dependency of *α* and the equivalent radiation dose: (**a**) Comparison of data obtained from the present study versus those obtained from a previous study to calculate the temperature dependency of *α*; (**b**) Comparison of the equivalent radiation dose escalation with different temperatures from the present and previous studies.

A numerical comparison of the equivalent radiation dose with values available in the literature^3^ was performed to validate the MATLAB code, as shown in Fig. 4. Temperatures of 40.5 °C, 41.6 °C, and 42.4 °C (minimum, mean, and maximum, respectively) were considered in the study, and enhancements in *α* from 0.0391 to 0.059 Gy^−1^ for 40.5 °C, 0.0615 Gy^−1^ for 41.6 °C, and 0.0654 Gy^−1^ for 42.4 °C were recorded, resulting in enhancement of the equivalent radiation doses. The equivalent radiation dose with RT alone was 60 Gy, whereas that of RT combined with HT increased to 70.3 Gy for 40.5 °C, 86.3 Gy for 41.6 °C, and 93.6 Gy for 42.4 °C. As shown in Fig. 4(a,b), the MATLAB code written for the calculation of the equivalent radiation dose in the present study reproduced the results of a previous study with similar input conditions, confirming the accuracy of the code and providing numerical validation.

**Figure 5:**
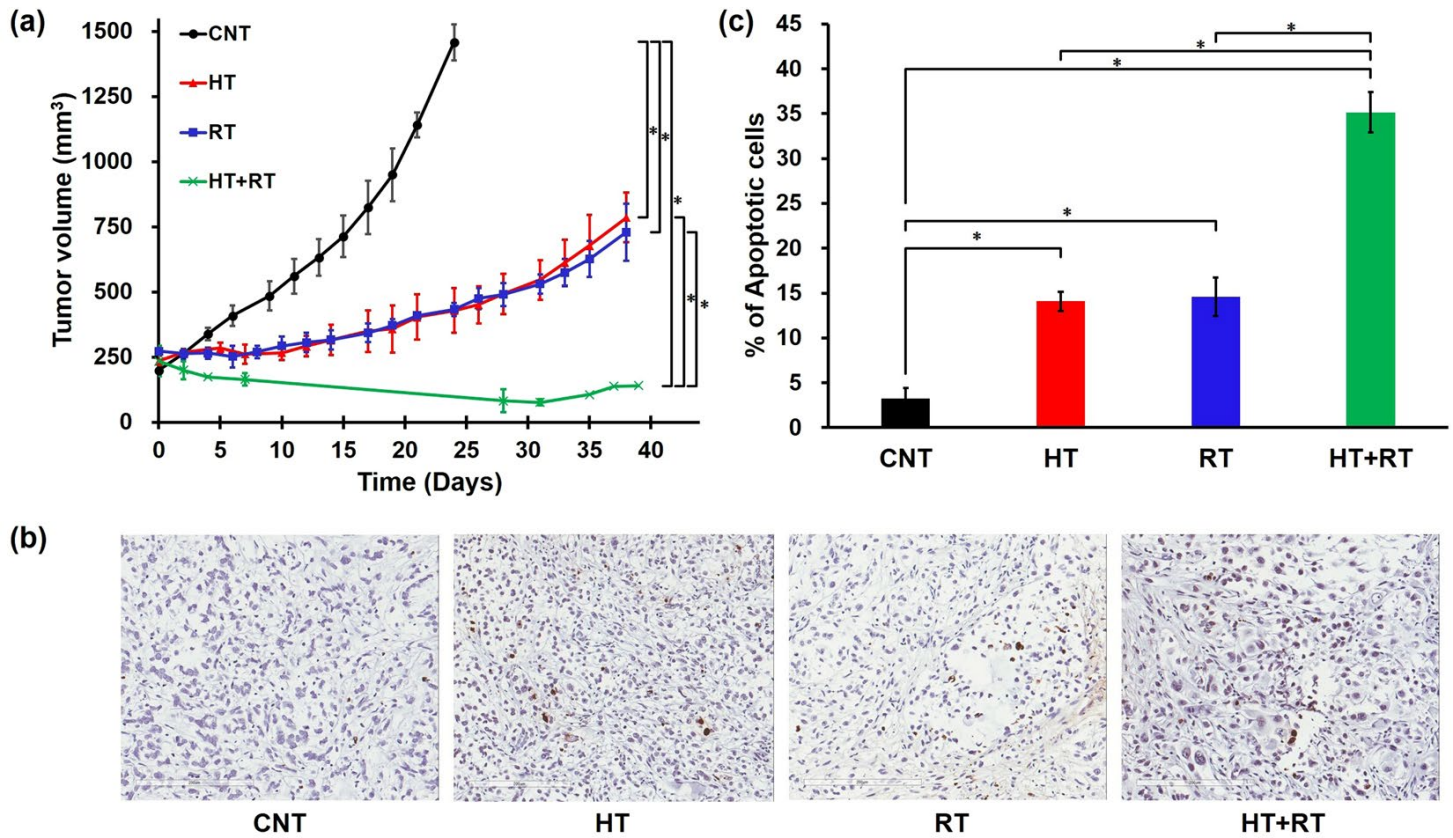
(**a**) Tumor growth curves of control (CNT), hyperthermia alone (HT), irradiation alone (RT), and hyperthermia followed by irradiation (HT + RT) groups (n = 3, *p < 0.05). Tumors of HT alone group were heated at 42 °C for 30 min at 2-day interval. Radiation dose was 5 Gy per fraction daily 2 fractions at 2-day interval. Tumors of HT + RT group were heated for 30 minutes at 42 °C prior to irradiation. Points: mean volumes of three tumors, bars: standard errors. (**b**) Apoptotic activity of each treatment group analyzed by terminal deoxynucleotidyl transferase-mediated deoxyuridine triphosphate nick end labeling (TUNEL) assay. TUNEL-positive nuclei were counted in 3~4 fields randomly selected per tumor sections (original magnification, ×200; scale bar, 200 µm). (**c**) Comparison of apoptotic rate of each treatment group (error bars, standard errors; *p < 0.05). Image analysis was performed using QuPath software.

The actual effect of combined HT and RT was compared with that of RT alone using mouse lung cancer xenografts. The tumor growth curves of control (CNT), HT alone (HT), RT alone (RT) and HT combined with RT (HT + RT) groups are shown in Fig. 5(a). Treatments were started when the tumor volume reached around 200~250 mm^3^. The volume of untreated CNT groups reached 2 times in 6 days while those of HT and RT groups increased 2 times in 27 and 32 days, respectively. The tumor volume of HT + RT groups didn’t reach 2 times until 42 days. The growth of tumors treated with HT + RT was significantly slower than that of tumors treated with HT or RT alone (p < 0.05). We examined whether apoptosis was associated with the antitumor effect of combination therapy. The apoptotic activity was measured by terminal deoxynucleotidyl transferase-mediated deoxyuridine triphosphate nick end labeling (TUNEL) staining (Fig. 5(b)). The percentage of apoptotic cells was significantly higher in combination therapy group than that of RT or HT alone group (Fig. 5(c), p < 0.05).

**Figure 6:**
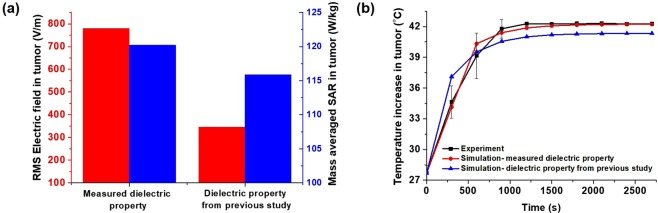
Comparison of electric field, energy absorption and temperature distribution: (**a**) Root mean square (RMS) electric field and the mass averaged specific absorption rate and (**b**) Temperature distribution obtained from the experiment and simulation of the mouse tumor model.

Figure 6 shows the quantitative estimation of the electric field, specific absorption rate (SAR) and increase in temperature in mouse tumors. These were found to be different with varying dielectric properties. Figure 6(a) Shows that the root mean square electric field and mass averaged SAR in the tumor with measured tumor dielectric properties in the present study was 780.3 versus 346.1 V/m and 120.2 versus 115.9 W/kg for dielectric properties measured in a previous study. The temperature achieved with the measured tumor dielectric properties in the present study was 42.2 °C versus 41.3 °C obtained from a previous study. This indicates that a variation in the dielectric properties may alter the temperature distribution in tumors, as shown in Fig. 6(b). The temperature obtained from thermal probes and simulation with measured dielectric properties in the present study was mapped well in contrast to that obtained from a previous study as shown in Fig. 6(b).

**Figure 7:**
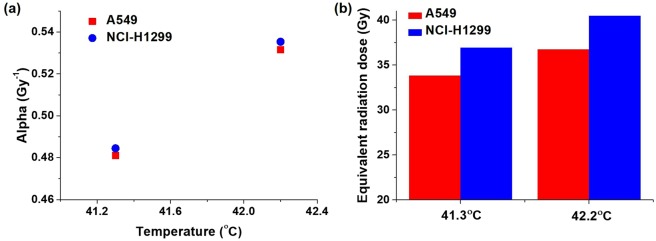
Comparison of the radiation dose escalation with temperature obtained from the dielectric properties measured in the present study and those obtained from a previous study: (**a**) effect on the radiosensitivity parameter *α*; (**b**) effect on the equivalent radiation dose.

Figure 7 Shows the comparison of the radiation dose escalation with temperature obtained from measurement of the dielectric properties in the present study versus those obtained from a previous study. The radiation dose was 20 Gy without hyperthermia, and with measured dielectric properties, the temperature increase in the tumor was 42.2 °C, and the corresponding enhanced equivalent radiation doses were 36.71 Gy for A549 cells and 40.44 Gy for H1299 cells. The temperature achieved from the simulation using the dielectric properties reported in a previous study was 41.3 °C, translating to an escalation of the *α* value to 0.48 Gy^−1^ for A549 cells and NCI-H1299 cells, an escalation of equivalent fraction dose to 2.94 Gy for A549 cells and 3.33 Gy for NCI-H1299 cells, and an escalation of equivalent radiation dose to 33.82 Gy for A549 cells and 36.90 Gy for NCI-H1299 cells. These findings are shown in Fig. 7(a,b). We found that the *α* values for A549 cells and NCI-H1299 cells at 41.3 °C were 0.05 Gy^−1^ lower than those observed at 42.2 °C. Consequently, the equivalent radiation doses were 2.89 and 3.54 Gy lower for A549 cells and NCI-H1299 cells, respectively, as shown in Fig. 7(b). These results suggest that tumor properties should be considered carefully for the accurate calculation of the equivalent radiation dose.

## Discussion

In the present study, the combined effect of RT and HT was determined through the calculation of the equivalent radiation dose using the linear temperature dependency of the *α*. The *α* and *β* parameters of radiation alone or combined with hyperthermia for lung cancer cell lines A549 and NCI-H1299 were obtained from experiments and the linear model for the temperature dependency of *α* was modified for lung cancer cells. The equivalent radiation dose escalation calculated for the NCI-H1299 xenograft was greater than that calculated for A549 tumors, indicating that radiation dose escalation during the addition of hyperthermia to the regimen may vary according to the different types of cancer.

The effect of combination treatment was verified with mouse xenograft model of A549 cells. HT before RT significantly enhanced the radiation-induced tumor growth delay. In a previous study with mouse fibrosarcoma, HT enhanced radiation-induced tumor growth delay. The radiosensitizing effect was more prominent when HT was applied before RT rather than after RT, and three times of repeated HT was more effective than single heating in suppressing tumor growth^13^. In our study, HT at 42 °C for 30 minutes was applied before each 5 Gy of RT and the combination treatment was more effective in suppressing tumor growth than HT or RT alone. HT was also found to enhance the apoptotic cell death of human lung cancer xenograft. Additive effect of HT and RT on the induction of apoptosis was shown by TUNEL assay (Fig. 5(c)). As for A549 tumors, similar effect may be obtained for NCI-H1299 xenografts. HT was reported to induce apoptosis of human hepatocellular carcinoma cells by up-regulating tumor suppressor SEPT4^14^. Increased cell death by combined HT + RT could be explained in part by increased apoptosis by HT.

In combined HT and RT treatment in clinics, addition of HT will change the homogeneity of tumor temperature from normothermic to hyperthermic range. This higher temperature distribution at mild hyperthermia range enhances the blood perfusion and thereby increases the oxygenation in tumor vessels which improves the efficacy of RT treatment^15^. Even though there is nonhomogeneous temperature distribution, previous studies reported that the T10, T50, and T90 (temperature achieved in 10, 50 and 90% of target tumor volume) were in the range 40–42 °C^3,5^. Therefore, most part of tumor could be in the hyperthermic range that has radiosensitizing effect, and these effects can be properly addressed with thermoradiotherapy planning in clinics.

*In vivo* measurement of temperature, along with electromagnetic and thermal simulations using mouse xenograft models, was performed to investigate the effect of dielectric property variation in temperature prediction and its corresponding equivalent radiation dose escalation. Studies reported that the dielectric properties of tumors vary according to the type and stage of cancer^16,17^, and this variation may predict inaccurate SAR and distribution of the temperature^18^. For the combination of RT with HT, the equivalent radiation dose may vary according to the variations in the dielectric properties of tumors. The dielectric properties of tumors were measured for present study, and the values reported previously at 13.56 MHz were used^19^ for comparison. Numerical simulations were performed to determine the SAR and temperature distribution using a mouse tumor model, comparing the dielectric properties of tumors measured in the present study with those obtained from a previous study. This comparison showed that the distribution was different^18^. When the calculated equivalent radiation dose was compared with the temperature achieved from two dielectric properties of the tumor and experimental measurement, the radiation dose escalation was underestimated from the predicted value.

Variations in the dielectric properties of tumors may either underestimate or overestimate the equivalent radiation dose escalation. Studies reported the dielectric property variation in liver and colorectal cancer on human tumors^16,17^. Patients with different type of liver tumor showed different dielectric property values with one patient having very large variation in permittivity and conductivity values^16^. A tumor property measurement on colorectal cancer considering different stages of cancer showed significant variations in the permittivity and conductivity values^17^. Hence, these variations should be considered carefully in the planning of clinical treatment. To improve the clinical treatment planning standards, noninvasive patient-specific dielectric property measurement techniques can be used, such as incorporation of magnetic resonance electrical properties tomography (MREPT)^20^ and dictionary‐based electric properties tomography (dbEPT)^21^.

In the validation study, the *α* and *β* values for prostate cancer obtained from a previous study were used^3^. Three temperature points (minimum, mean, and maximum) obtained in the tumor tissues and their corresponding equivalent radiation doses were considered in this study. The results were in agreement with the MATLAB code produced for the present study. The linear model for temperature dependency in the validation study was determined using the equation provided in a previous study, and the equivalent radiation dose escalation was calculated by multiplying the equivalent fractional dose by the number of fractions^3^. For the lung cancer cell lines used in the present study, the linear equation was modified based on the cell experiment, and the calculations of the equivalent radiation dose were performed using the Lea–Catcheside protraction factor^4^.

An accurate identification of radiosensitivity parameters and energy-source-dependent tissue properties is necessary for thermoradiotherapy treatment planning. In the present study, the radiofrequency (RF) energy source was used; thus, the tumor-specific dielectric properties were important. If thermoradiotherapy is performed using other energy sources such as laser or ultrasound, the absorption coefficient and the acoustic properties of the tumor tissues should be considered carefully for the accurate estimation of radiation dose escalation.

The present study uses mathematical models based on previous studies^3,4^ for the calculation of the equivalent radiation dose. Further modifications may be required for determining the radiation dose escalation with high precision for lung cancer. However, the trend in the radiation dose escalation may be similar to that reported in the present and previous studies^3,5^. A modified linear model is proposed to determine the thermal sensitivity of lung cancer cell lines A549 and NCI-H1299. However, further studies are warranted to derive a general formulation applicable to the treatment planning for lung cancer using the combination of RT with HT. In addition, studies considering the exponential increment of *α*, thermal dose threshold temperatures, time dependency, and the effect of perfusion or ventilation on tumor temperature are warranted to develop lung-cancer-specific treatment planning strategies in clinical practice.

## Conclusion

The combined effects of RT and HT were quantified in terms of the equivalent radiation dose. The thermal enhancement of the radiosensitivity parameters of lung cancer cell lines A549 and NCI-H1299 was determined. Moreover, the estimated equivalent radiation doses were escalated using hyperthermia in both cell lines. The effect of enhanced equivalent radiation dose was confirmed with mouse xenograft models. The effect of the variation in the dielectric properties of tumors was also determined. The results showed that inaccurate estimations of the dielectric properties of tumors may lead to underestimation of the equivalent radiation doses. Therefore, the variation of the dielectric properties of tumors should be considered carefully in the planning of RF-induced thermoradiotherapy.

## Materials and Methods

### *In vitro* cell culture and treatment

The human lung carcinoma cell lines A549 and NCI-H1299 were purchased from the Korean Cell Line Bank (Seoul, Korea). The cells were cultured in RPMI 1640 (HyClone, South Logan, UT, USA) supplemented with 10% inactivated fetal bovine serum (HyClone) at 37 °C in an atmosphere of 5% CO_2_.

The LAB-EHY100 (OncoTherm, Budapest, Hungary) device was used for the hyperthermia treatment. The cells were placed in the heating chamber (LAB-EHY *in vitro* applicator) with culture medium at 42 °C for 30 min. Irradiation of cells was performed using a ^137^Cs gamma-irradiator (MK 1-68, JL Shepherd, San Fernando, CA, USA) at a rate of 2.75 Gy/min.

The A549 and NCI-H1299 cells were plated into 60-mm dishes and exposed to 0, 2, 4, and 8 Gy radiation. For the combination treatment, cells were treated using the LAB-EHY100 at 42 °C for 30 min prior to irradiation. After 14 days of incubation, the colonies were stained with crystal violet, and those with more than 50 cells were counted. The plating efficiency of the control group and the surviving fraction of each treatment group were calculated. All experiments were performed in triplicate. The cell survival curve was fitted to the  LQ model for the calculation of radiobiological parameters.

### *In vivo* mouse experiment

BALB/c nude mice were obtained from Koatech (Gyeonggi-do, Korea). The A549 and NCI-H1299 cells (5 × 10^6^/100 μL) were subcutaneously injected into the right hind leg of the nude mice. After 1–2 months, the tumors were detected in the mice. The tumor volumes were calculated using the formula *V* = *(π* × *L* × *W* × *H)*/6, with *L, W*, and *H* representing the length, width and height of tumor in mm, respectively.

Hyperthermia treatment (HT) in the mouse xenograft model was performed using the 13.56 MHz radiofrequency (RF) capacitive heating device LAB-EHY 100 as shown in Fig. 1(a) to determine the increase in temperature (41–42 °C) in the tumors^13,18^. Fiberoptic sensors were inserted into the tumor to determine the increase in temperature during RF heating. The tumor was heated to 42 °C and maintained at this temperature for 30 min.

The tumor growth delay was compared among HT or irradiation (RT) alone group and irradiation following hyperthermia (HT + RT) group using A549 xenografts. Mice of HT alone group received 2 times of HT at 2-day interval as described above. For irradiation, mice were immobilized in jigs with prone position and the tumors on the right hind legs were irradiated with a linear accelerator (Varian 21EX^®^, Varian Medical Systems, Palo Alto, CA, USA). 5 Gy per daily dose of radiation was delivered twice at 2-day interval. For mice in the HT + RT group, hyperthermia was followed by 5 Gy of radiation within 4 hours and the combination treatment was repeated at 2-day interval. Each group consisted of 3 mice.

Apoptotic activity was analyzed on the basis of TUNEL assay. Tumors from each group of three tumor-bearing mice, apart from the mice used for tumor growth study, were excised 3~5 days after treatment. For histologic detection, tumor samples were fixed in 10% neutral formalin and embedded in paraffin. The histological sections were subjected to TUNEL staining using an *in situ* ApopTag^®^ kit (Millipore, Temecula, CA, USA) for apoptosis detection. The percentage of TUNEL-positive cells were calculated at 3~4 random area per sample. Image of tissue section was analyzed using QuPath, an open-source software for quantitative pathology^22^.

The experimental protocol used in this study was approved by the Institutional Animal Care and Use Committee (17-0110-S1A1, Seoul National University Hospital) and performed in compliance with the committee guidelines and regulations. A detailed description of the experimental procedures performed in this study has been provided in our previous research^18^.

### Dielectric property measurement of tumor

The dielectric properties of tumors were measured using an impedance analyzer (E4991A; Agilent Technologies, Santa Clara, CA, USA), and the details of the measurement and analysis of the dielectric properties have been described in our previous research^18^.

### Electromagnetic and thermal simulations

Electromagnetic and thermal simulations were performed using the multiphysics simulation platform Sim4Life (Zurich Med Tech, Zurich, Switzerland)^23–25^. A three-dimensional mouse model, reconstructed from computed tomography images, was used for the simulations as shown in Fig. 1(b) ^18,26^. Dielectric and thermal properties of tumor and other body sites are listed in Table 3 ^18,27^. The governing equations used for the electromagnetic simulations based on quasi-static approximation are provided below^23,28–30^:

1 $$\nabla (\,-\,\varepsilon \nabla \varphi )=0,\,E=-\,\nabla \varphi $$

2 $$SAR=\frac{\sigma }{2\rho }{|E|}^{2}$$

3 $${Q}_{r}=\rho SAR=\frac{\sigma }{2}{|E|}^{2}$$

**Table 3:** Dielectric (13.56 MHz) and thermal properties of tissues used in the simulations.

Material	Density (kg/m^3^)	Electric Conductivity (S/m)	Relative Permittivity	Thermal Conductivity (W/m·K)	Specific Heat (J/kg·K)	Perfusion (mL/min/kg)	Heat Generation (W/kg)
Skin	1109	0.238	285.24	0.3721	3390.5	106.38	1.64
Rectum	1088	0.512	217.11	0.542	3657.5	786.23	11.85
Bone	1908	0.045	30.57	0.32	1312.8	10	0.15
Mouse Tumor (previous study)	1070	0.683	266.99	0.4949	3421.2	18.36	0.9
Mouse Tumor (measured)	1070	0.7847	278.85	0.4949	3421.2	18.36	0.9
Distilled Water	1000	0.00005	76.7	0.563	4181.3	—	—

In the equation above, *ε* is the permittivity, *φ* is the electric potential, *E* is the electric field strength, *SAR* is the specific absorption rate, *σ* is the electrical conductivity, *ρ* is the mass density, and *Q*_*r*_ is the heat source.

For the thermal simulation, Pennes’ bio-heat transfer model was used^31^. The energy obtained from the electromagnetic simulation is provided as a user-defined heat source.

4 $$\rho c\frac{\partial T}{\partial t}=\nabla \cdot (k\nabla T)-{\rho }_{b}{c}_{b}{\omega }_{b}(T-{T}_{b})+{Q}_{r}+{Q}_{m}$$

In the equation above, *c* is the specific heat, *T* is the temperature, *t* is the time, *k* is the thermal conductivity, *Q*_*m*_ is the metabolic heat-generation rate, *ω*_*b*_ is the perfusion rate, and *ρ*_*b*_, *c*_*b*_, and *T*_*b*_ correspond to the density, specific heat, and temperature of blood, respectively. The thermal properties, perfusion, and generation of metabolic heat used for the simulation are listed in Table 3 ^3,27^. A convective boundary with a surface heat transfer coefficient of 5 W/m^2^K and an ambient temperature of 25 °C were used as the boundary conditions. An initial temperature of 27.7 °C was applied to all body sites, and the duration of the simulation was 45 min. Grid independent studies were conducted and an optimum mesh of 1.98 M cells are used for the simulation.

### Calculation of the equivalent radiation dose

A MATLAB code was used for the calculation of the equivalent radiation dose based on the linear–quadratic (LQ) model^32^. The LQ model may be expressed as^3^

5 $$SF(n,d,\alpha ,\beta ,T)={e}^{-n(\alpha (T)d+\beta {d}^{2})}$$

In the equation above, *SF* is the cell surviving fraction, *d* is the fractionated dose, *n* is the number of fractions, *α* and *β* are the radiosensitivity parameters, and *T* is the temperature.

Hyperthermia may alter *α* and *β*. In general, changes are more pronounced in *α* than in *β* because *α* is thought to represent the subset of DNA repair mechanisms. Hence, only an enhancement in *α* was considered in the present study. Temperature dependencies of *α* may be calculated using a linear model^3^. Considering the baseline temperature in this experiment and the thermal sensitivity of the lung cancer cells obtained from the experiments, the linear model was modified to provide an accurate estimation.

6 $$\alpha (T)={\alpha }_{37}+\frac{2.17{\alpha }_{37}-{\alpha }_{37}}{42-37}(T-37)$$

The equivalent fraction dose with hyperthermia (*d*_*HT*_), independent of the number of fractions, may be expressed as^3^

7 $${d}_{HT}=\frac{-{\alpha }_{37}+\sqrt{{{\alpha }_{37}}^{2}+4{\beta }_{37}d(\alpha (T)+d{\beta }_{37})}}{2{\beta }_{37}}$$

The equivalent radiation dose in hyperthermia (*EQD*_*RT*_) for external beam radiotherapy considering the Lea–Catcheside protraction factor may be calculated using the following equation^4^:

8 $$EQ{D}_{RT}=\frac{\alpha (T)D+G{\beta }_{37}{D}^{2}}{{\alpha }_{37}+{\beta }_{37}d}$$

In the equation above, *D* is the total radiation dose, and *G* is the Lea–Catcheside protraction factor. The input parameters used for the calculation of the equivalent radiation dose escalation are listed in Table 2.

### Statistical analysis

*In vitro* or *in vivo* experimental data were expressed as mean ± standard error of mean. The statistical differences were assessed with Student’s *t*-test. A threshold of *p* < 0.05 was defined as statistically significant.
